# Reimagining ion-transport pathways for all-solid-state lithium batteries

**DOI:** 10.1093/nsr/nwag123

**Published:** 2026-03-05

**Authors:** Yu Wang, Hui Pan, Di Zhang, Bao-Lian Su

**Affiliations:** State Key Laboratory of Metal Matrix Composites, School of Materials Science and Engineering, Shanghai Jiao Tong University, China; Laboratory of Inorganic Materials Chemistry, University of Namur, Belgium; State Key Laboratory of Metal Matrix Composites, School of Materials Science and Engineering, Shanghai Jiao Tong University, China; State Key Laboratory of Metal Matrix Composites, School of Materials Science and Engineering, Shanghai Jiao Tong University, China; Laboratory of Inorganic Materials Chemistry, University of Namur, Belgium; State Key Laboratory of Advanced Technology for Materials Synthesis and Processing, Wuhan University of Technology, China

All-solid-state lithium batteries (ASSLBs) stand as a transformative vision for next-generation energy storage, seamlessly integrating high energy density with intrinsic safety [1]. The relentless pursuit of advanced ASSLBs has driven rapid progress in lithium-ion solid-state electrolytes (SSEs), yet commercialization remains severely bottlenecked: no existing SSEs can simultaneously achieve fast ion transport, a broad electrochemical stability window and robust environmental stability [2].

Current SSE research encompasses four primary categories: sulfides, oxides, halides and polymer-based electrolytes [3], and each of these exhibits distinct strengths and inherent limitations [4,5]. Sulfide-based SSEs boast room-temperature ionic conductivities comparable to those of liquid electrolytes (10^−^^2^ S cm^−1^), but their extreme moisture sensitivity and narrow electrochemical window impede practical deployment. Oxide SSEs excel in electrochemical stability yet suffer from low-room-temperature ionic conductivity, restricting performance under ambient conditions. Halide SSEs have reemerged as promising candidates due to moderate ionic conductivity and favorable cathode compatibility, but persistent challenges in interfacial stability and the electrochemical window limit their application. A fundamental constraint across all conventional SSEs lies in their single-anion frameworks [6]. Innovative mixed-anion framework designs could address these intrinsic limitations, enabling versatile SSEs with balanced high ionic conductivity and stable interfaces, thereby accelerating the development of next-generation ASSLBs.

**Figure 1.**
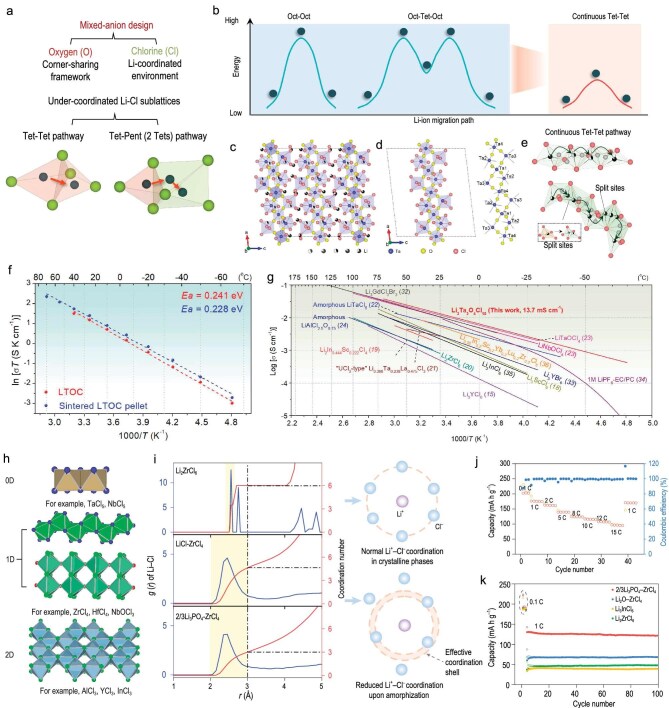
Schematic illustrations and performance characterizations of mixed-anion and solid dissociation-based SSEs. (a) Mixed-anion strategy framework. (b) Schematic illustration of Li-ion migration energy along different pathways. (c) Crystal structure of the LTOC along the *b*-axis. (d) Ta-O/Cl long chain extracted from LTOC. (e) Continuous LiCl*_x_* (*x* = 4, 5) polyhedra forming the Li sublattices in LTOC. (f) Arrhenius plots of Li-ion transport. (g) Ionic-conductivity comparison between representative halide-based SSEs. (h) Low-dimensional building blocks of (oxy)chloride van der Waals (vdW) crystals. (i) Pair distribution function (*G*(*r*)) extracted from *ab initio* molecular dynamics (AIMD) simulations. (j) Rate performance and (k) cycling stability of cells incorporating solid dissociation-based electrolytes. Adapted with permission from Sun *et al.* [7,8].

Writing in *Science*, Sun and colleagues proposed an atomic-level strategy for engineering anion sublattices to achieve superionic conduction [7]. By leveraging a mixed-anion approach, they developed the crystalline oxychloride Li_3_Ta_3_O_4_Cl_10_ (LTOC), featuring a framework of corner-sharing polyhedra in which oxygen and chlorine anions are used to construct under-coordinated Li–Cl sublattices (Fig. 1a). This configuration generates unique lithium-ion migration pathways, notably tetrahedral–tetrahedral (tet–tet) channels confined to a bcc-like anion sublattice—a structural motif that is absent from conventional oxide or halide chemistries (Fig. 1b). Energy calculations and atomic-scale simulations further validated that these tet–tet pathways bypass the high-energy barriers typical of traditional octahedral sites, enabling exceptionally low activation energies (0.14–0.28 eV) and facilitating rapid 3D lithium transport (Fig. 1c–e).

Experimentally, the LTOC electrolyte exhibits a record-high room-temperature ionic conductivity of 13.7 mS cm^−1^ and electrochemical stability, surpassing those of conventional sulfide (Li_6_PS_5_Cl) and halide (Li_3_YCl_6_) electrolytes (Fig. 1f). More importantly, this work provides direct evidence that mixed-anion sublattice engineering can overcome the long-standing trade-off between fast ion transport and electrochemical stability in SSEs. The crystalline lattice of LTOC further exhibits high tolerance to cation and anion doping, reflecting an unusual structural flexibility that enables compatibility with lithium metal and offers practical latitude for compositional tuning, cost reduction and scalable synthesis. Temperature-dependent conductivity measurements further confirm the stable performance of LTOC across a wide temperature range, highlighting its potential under extreme operating conditions (Fig. 1g)—a critical attribute for sustaining performance under realistic battery cycling. Full cells that incorporate LTOC as the electrolyte achieve stable long-term cycling at high rates (3C, >4000 cycles) and retain exceptional capacity even at ultralow temperatures (−50°C, 0.1C, >2000 cycles), demonstrating its practical viability.

Building on these foundational insights, Sun and co-workers extended the mixed-anion conceptual framework beyond crystalline design, introducing a pioneering solid-dissociation strategy in *Nature Energy* [8]. Recognizing that crystalline frameworks are often constrained by dopant–lattice compatibility, they proposed using halide van der Waals materials (e.g. InCl_3_ or ZrCl_4_) as solid solvents to dissolve various inorganic salts (Fig. 1h). This concept establishes a microscopic analogy to the fundamental role of liquid electrolytes, in which internal

structural rearrangements disrupt the salt lattice and ultimately generate a fully disordered amorphous matrix with intrinsically high ionic conductivity. Pair distribution function analysis, ^7^Li solid-state nuclear magnetic resonance and *ab initio* molecular dynamics simulations revealed the formation of a Li-bond-rich environment originating from under-coordinated, distorted LiCl*_x_* (*x* = 3–5) configurations (Fig. 1i), providing high-flux pathways for rapid cation transport. This platform enables precise compositional tuning, achieving high-voltage stability (≤4.8 V) and demonstrating the versatile conduction of Li^+^, Na^+^, Ag^+^ and Cu^+^. Additionally, electrolytes suitable for 15C rate charge/discharge were developed (Fig. 1j), exhibiting high oxidative and air stability (Fig. 1k).

Despite these significant advances, critical challenges persist in translating laboratory breakthroughs into industrial-scale deployment. Although mixed-anion strategies enhance bulk ionic conductivity, achieving durable chemo-mechanical stability at solid–solid interfaces remains difficult, particularly under the repeated volume changes of high-capacity anodes. Moreover, the dynamic evolution of Li-bond-rich environments in amorphous systems at high current densities remains poorly understood and requires advanced *operando* probes.

In summary, these studies demonstrate that mixed-anion sublattice engineering can decouple ionic mobility from electrochemical and environmental stability, overcoming the fundamental limitations of single-anion electrolytes. By establishing continuous, low-energy tet–tet migration pathways, this strategy introduces a new structural design principle for SSEs with balanced transport and stability. Such advances mark a critical step toward practical ASSLBs capable of reliable operation under diverse and extreme conditions, setting a benchmark for innovation in materials chemistry and energy-storage research.
